# Studies on Bd0934 and Bd3507, Two Secreted Nucleases from *Bdellovibrio bacteriovorus*, Reveal Sequential Release of Nucleases during the Predatory Cycle

**DOI:** 10.1128/JB.00150-20

**Published:** 2020-08-25

**Authors:** Ewa Bukowska-Faniband, Tilde Andersson, Rolf Lood

**Affiliations:** a Department of Clinical Sciences Lund, Division of Infection Medicine, Lund University, Lund, Sweden; University of Illinois at Urbana Champaign

**Keywords:** *Bdellovibrio bacteriovorus*, predatory bacteria, predatosome, secreted nucleases

## Abstract

Bdellovibrio bacteriovorus is an obligate predatory bacterium that invades and kills a broad range of Gram-negative prey cells, including human pathogens. Its potential therapeutic application has been the subject of increased research interest in recent years. However, an improved understanding of the fundamental molecular aspects of the predatory life cycle is crucial for developing this bacterium as a “living antibiotic.” During intracellular growth, B. bacteriovorus secretes an arsenal of hydrolases, which digest the content of the host cell to provide growth nutrients for the predator, e.g., prey DNA is completely degraded by the nucleases. Here, we have, on a genetic and molecular level, characterized two secreted DNases from B. bacteriovorus, Bd0934 and Bd3507, and determined the temporal expression profile of other putative secreted nucleases. We conclude that Bd0934 and Bd3507 are likely a part of the predatosome but are not essential for the predation, host-independent growth, prey biofilm degradation, and self-biofilm formation. The detailed temporal expression analysis of genes encoding secreted nucleases revealed that these enzymes are produced in a sequential orchestrated manner. This work contributes to our understanding of the sequential breakdown of the prey nucleic acid by the nucleases secreted during the predatory life cycle of B. bacteriovorus.

**IMPORTANCE** Antibiotic resistance is a major global concern with few available new means to combat it. From a therapeutic perspective, predatory bacteria constitute an interesting tool. They not only eliminate the pathogen but also reduce the overall pool of antibiotic resistance genes through secretion of nucleases and complete degradation of exogenous DNA. Molecular knowledge of how these secreted DNases act will give us further insight into how antibiotic resistance, and the spread thereof, can be limited through the action of predatory bacteria.

## Abstract (precis)

Antibiotic resistance is a major global concern with few available new means to combat it. From a therapeutic perspective, predatory bacteria constitute an interesting tool. They not only eliminate the pathogen but also reduce the overall pool of antibiotic resistance genes through secretion of nucleases and complete degradation of exogenous DNA. Molecular knowledge of how these secreted DNases act will give us further insight into how antibiotic resistance, and the spread thereof, can be limited through the action of predatory bacteria.

## INTRODUCTION

**FIG 1:**
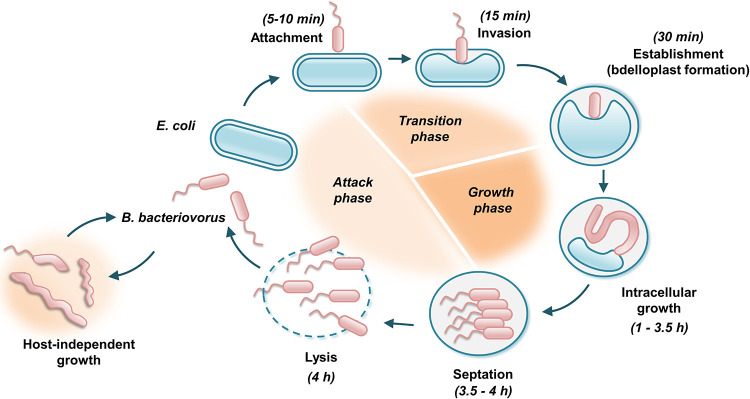
Life cycle of B. bacteriovorus (for a detailed description see the text).

Bdellovibrio bacteriovorus is the most studied species among predatory bacteria and serves as a model for understanding the unique cell biology of bacterial predators. It invades and kills a broad range of Gram-negative bacteria, including human pathogens such as Acinetobacter baumannii, Klebsiella pneumoniae, and Escherichia coli (1, 2). The B. bacteriovorus life cycle consists of three stages: attack phase, transition phase, and growth phase (Fig. 1) (3–5). In the attack phase, the organism is free-swimming and moving around in search of prey. Upon collision with a prey cell, it attaches itself to the outer membrane and assesses the prey’s nutritional value (transition phase). When the right prey is recognized, its cell surface is modified so that the predator can pass through without destroying it. Once in the periplasm, *Bdellovibrio* forms a structure known as the bdelloplast and transitions into growth phase. The cytoplasmic content of the prey is degraded by an arsenal of predatory hydrolytic enzymes released into the bdelloplast milieu (6, 7). The resulting nutrients are subsequently taken up and used for filamentous growth of the predator. When resources are exhausted, *Bdellovibrio* divides, and the progeny lyse the remains of the cell. The complete life cycle takes 3 to 4 h under laboratory conditions. Despite being considered an obligate predator, B. bacteriovorus can also convert into a host-independent lifestyle (8).

The breakdown of prey substrates and their subsequent utilization appear to be highly organized events that proceed in a sequential manner (9–11). However, the kinetics of biochemical processes may vary depending on the prey species (11). When B. bacteriovorus is grown on E. coli, degradation of the host DNA into fragments of intermediate molecular weight is achieved within the first 45 to 60 min of infection. This rapid step is apparently followed by slow degradation into mononucleotides, which are used for the synthesis of *Bdellovibrio* DNA. It is estimated that approximately 70% of host DNA is incorporated into the newly synthesized DNA of the predator (9, 11).

Although it is not known precisely which enzymes are involved in the digestion of host genetic material, it has been demonstrated that host intrinsic nucleases are not involved in this process (9). The B. bacteriovorus HD100 genome encodes 20 putative DNases, some with an apparent export signal sequence (7). A transcriptome-based approach identified two DNases that are upregulated upon contact with the prey cell, Bd1244 and Bd1934 (6). However, genetic studies by Lambert and Sockett demonstrated that these two DNases are not essential for the degradation of host DNA (12). Based on the presence of a signal peptide, two other putative DNases have been suggested to have a function in this process, Bd0934 and Bd3507. However, due to the lack of the apparent upregulation upon contact with the prey (6), they were considered less likely to be involved in the host DNA breakdown and have not been characterized until now.

In this paper, we characterize Bd0934 and Bd3507 in terms of expression profile over the life cycle, subcellular localization, importance for predation ability, host-independent growth, self-biofilm formation, and prey biofilm predation. We also determine the full transcription profile of other putative secreted nucleases. The results show that during intraperiplasmic growth, the B. bacteriovorus nucleases are released in an orchestrated sequential manner. Bd0934 and Bd3507 are secreted into the bdelloplast milieu and seem to act in the midstages of prey infection.

## RESULTS

### *bd0943* and *bd3507* are expressed during the intracellular growth phase.

**FIG 2:**
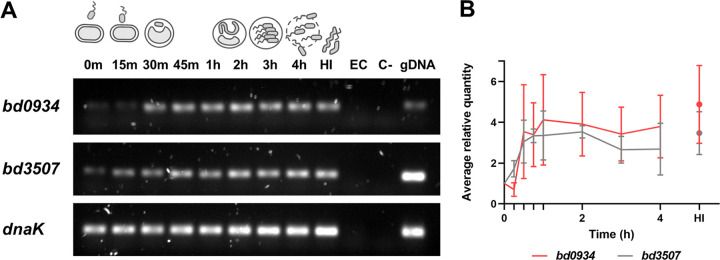
(A) Semi-qRT-PCR analysis to assess expression of *bd0934* and *bd3507* throughout the B. bacteriovorus HD100 life cycle. Samples for RNA isolation were collected from semisynchronous predatory coculture at the indicated time points. A matched amount of RNA (10 ng) was used as a template in RT-PCRs. Equal volumes of each RT-PCR sample were analyzed by 1.5% agarose gel electrophoresis. *dnaK* is constitutively expressed in both host-dependent and host-independent growth and, thus, serves as an internal reference gene. HI, sample with RNA isolated from host-independent strain LHI100 used as a template; EC, sample with RNA isolated from E. coli DH5α used as a template (negative control); C-, sample with no template (negative control); gDNA, sample with genomic DNA from B. bacteriovorus used as a template (positive control). A representative gel electrophoresis of three independent experiments is shown. (B) *bd0934* and *bd3507* expression levels quantified relative to *dnaK*. The average values ± SD from three independent semi-qRT-PCR experiments are plotted.

A previous transcriptomic study showed that neither *bd0934* nor *bd3507* was upregulated in the early stage of infection, i.e., at 30 min into the predatory cycle (6). Instead, results implied that these two genes were specifically expressed during host-independent growth. To get insight into the full expression profile of *bd0943* and *bd3507*, we carried out a semiquantitative reverse transcription PCR (semi-qRT-PCR) analysis of samples collected throughout the B. bacteriovorus HD100 life cycle. As shown in Fig. 2, transcripts for both genes appear early in the predatory cycle and continue to increase in abundance until approximately 1 h postinfection. Both transcripts are present until the end of the intracellular growth cycle. The levels of expression in host-independent cells are similar to the levels present in the parasitic growth phase. These data indicate that Bd0934 and Bd3507 are involved in the degradation of host DNA in the bdelloplast and have a function in host-independent growth.

### Deletion of *bd0934* and *bd3507* does not affect predation.

To further investigate the role of Bd0934 and Bd3507, Δ*bd0934* (named LHD104) and Δ*bd3507* (named LHD103) single-deletion mutant strains were constructed (Table 1). The entire open reading frames (except the start and the stop codons) of the two genes were replaced by the chloramphenicol cassette as described in Materials and Methods. Out of 13 plaques screened by PCR for the *bd3507* deletion, 3 contained the desired mutation. In the case of *bd0934*, only 1 out of 71 screened plaques was found to be a successful deletion mutant. Predation efficiency of the obtained strains was assayed by predatory kill curves of semisynchronous cultures containing E. coli prey. Results showed that predation ability was not affected in any of the single-deletion strains compared to that of the wild type (see Fig. S1A in the supplemental material). Moreover, LHD103 and LHD104 strains yielded progeny titers similar to those of the wild type (∼2 × 10^8^ to 3 × 10^8^ PFU/ml) and had normal cell morphology, as determined by the phase-contrast microscopy (data not shown). Since no obvious phenotype was observed for the single-deletion strains, a Δ*bd0934* Δ*bd3507* double-deletion strain was constructed by introducing a markerless in-frame deletion of the *bd3507* gene into B. bacteriovorus LHD104. A PCR-based screen showed that 1 out of 70 analyzed plaques was a successful double-deletion mutant (named LHD110) (Table 1). Similar to the single-deletion strains, LHD110 did not exhibit a change in predation (Fig. S1A), progeny yield, or cell morphology (data not shown).

**TABLE 1:** Strains and plasmids used in this work

Strain or plasmid	Genotype or description^*a*^	Origin/reference
E. coli		
DH5α	F^–^ *endA1 glnV44 thi-1 recA1 relA1 gyrA96 deoR nupG purB20* ϕ80d*lacZ*ΔM15 Δ(*lacZYA-argF*)*U169 hsdR17(*r_K_*^–^* m_K_*^+^) λ^–^*	Invitrogen
S17-1	*recA pro hsdR* RP4­2­Tc::Mu­Km::Tn*7*	DSMZ^*b*^
TOP10	F^–^ *mcrA* Δ(*mrr-hsdRMS-mcrBC*) ϕ80*lacZ*ΔM15 Δ*lacX74 recA1 araD139* Δ(*ara-leu*)*7697 galU galK λ^–^ rpsL*(Str^r^) *endA1 nupG*	Invitrogen
B. bacteriovorus		
HD100	Wild type	DSMZ
LHD101	*bd0934*ΩpEBF1; Km^r^ Cm^r^	This work
LHD102	*bd3507*ΩpEBF2; Km^r^ Cm^r^	This work
LHD103	*bd3507*::cm Cm^r^	This work
LHD104	*bd0934*::cm Cm^r^	This work
LHD110	*bd0934*::cm Δ*bd3507* Cm^r^	This work
LHI100	HD100 host-independent spontaneous mutant	This work
LHI105	LHD103 host-independent spontaneous mutant	This work
LHI106	LHD104 host-independent spontaneous mutant	This work
LHI112	LHD110 host-independent spontaneous mutant	This work
Plasmids		
pFW13	Plasmid containing kanamycin resistance gene; Km^r^	34
pUC19	Cloning vector; Ap^r^	Invitrogen
pK18mobsacB	Mobilizable suicide vector, with *sacB* gene facilitating sucrose counterselection; Km^r^	ATCC^*c*^ (35)
pPROBE-NT	Broad-host-range vector containing promotorless *gfp* reporter gene; Km^r^	Addgene (36)
pEBF1	pK18mobsacB with 2.2-kb insert containing the Cm cassette flanked by 0.7-kb regions upstream and downstream of *bd0934*; Km^r^ Cm^r^	This work
pEBF2	pK18mobsacB with 2.2-kb insert containing the Cm cassette flanked by 0.7-kb regions upstream and downstream of *bd3507*; Km^r^ Cm^r^	This work
pEBF5	pEBF2 with deleted Cm cassette; Km^r^	This work
pEBF6	pPROBE-NT with 1.6-kb insert containing P_bd0934_-*bd0934*-mCherry; Km^r^	This work
pEBF7	pPROBE-NT with 2.0-kb insert containing P_bd3507_-*bd3507*-mCherry; Km^r^	This work
pEBF11	pPROBE-NT with 1.0-kb insert containing P_bd3507_-mCherry; Km^r^	This work

a Ap^r^, Cm^r^, and Km^r^ indicate resistance to ampicillin, chloramphenicol, and kanamycin, respectively.

b German Collection of Microorganisms and Cell Cultures, Germany.

c American Type Culture Collection, USA.

Seeing no effect of Bd0934 and Bd3507 deficiency in host-dependent cells, we isolated spontaneous host-independent derivatives of the Δ*bd0934* strain (named LHI106), the Δ*bd3507* strain (named LHI105), and the Δ*bd0934* Δ*bd3507* strain (named LHI112) (Table 1) and analyzed their axenic growth in PY medium. Host-independent derivatives isolated from the same parental strain are known to exhibit diverse phenotypes (13); thus, two individual host-independent isolates of each strain were tested in all the experiments described in this paper. The growth curves and phase-contrast microscopy data showed no apparent difference between the host-independent wild-type and nuclease deletion strains (Fig. S1B; microscopy data not shown). Thus, together these results indicate that Bd0934 and Bd3507 are not essential for host-dependent or for host-independent growth of B. bacteriovorus.

### Bd0934 and Bd3507 are secreted extracellularly.

**FIG 3:**
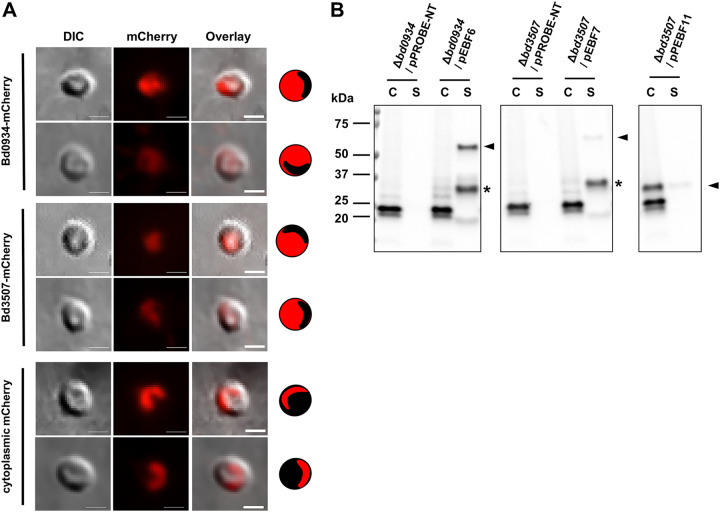
Extracellular localization of Bd0934-mCherry and Bd3507-mCherry, as determined by fluorescence microscopy and immunoblotting. (A) Fluorescence images of samples collected at 2 h into the predatory cycle. Bd0934-mCherry (strain LHD104/pEBF6) and Bd3507-mCherry (strain LHD103/pEBF7) localize to the bdelloplast milieu. The control strain, encoding only mCherry (strain LHD103/pEBF11), shows localization of the fluorescent protein in the cytoplasm of *Bdellovibrio* cells. Cartoon interpretation of the fluorescence image is depicted on the right side of each panel. Sixty bdelloplasts were observed for strain LHD104/pEBF6, 89 bdelloplasts were observed for strain LHD103/pEBF7, and 43 bdelloplasts were observed for strain LHD103/pEBF11. Representative bdelloplasts are shown. The dark shadow visible at the bottom left of the bdelloplasts in the DIC channel is an optical artifact. Scale bars are 1 μm. (B) Immunoblot of samples collected from host-independent B. bacteriovorus cultures (OD_600_ of ∼0.4) and separated into cell (C) and supernatant (S) fractions. An equal volume (15 μl) of each fraction was analyzed. pEBF6 encodes Bd0934-mCherry (∼55 kDa); pEBF7 encodes Bd3507-mCherry (∼59 kDa); pEBF11 encodes cytoplasmic mCherry (∼27 kDa); pPROBE-NT is an empty vector that serves as a negative control for nonspecific binding of antibodies. Immunoblotting was conducted against mCherry. The position of full-length proteins is indicated by an arrowhead. An asterisk indicates mCherry that is cleaved off the fusion protein. The antigen band at ∼25 kDa that is seen in all C fractions is a background independent of mCherry. Molecular mass markers are indicated on the left.

Both Bd0934 and Bd3507 have a predicted signal peptide sequence at the N-terminal end (Table S2). Therefore, they can be localized in the B. bacteriovorus periplasm or secreted out from the predator cell. To determine their subcellular localization, the two DNases have been fused to mCherry at the C-terminal end. The expression of *bd0934-mCherry* and *bd3507-mCherry* gene fusions was controlled by the native promoter of the respective nuclease. We first looked at subcellular localization during the host-dependent life cycle. For that purpose, fluorescence microscopy was employed, and cells were imaged 2 h postinfection. For both protein fusions, mCherry fluorescence was detected within the bdelloplast but outside the B. bacteriovorus growing cell (Fig. 3A). Such localization indicates that Bd0934-mCherry and Bd3507-mCherry are secreted out from the predator cell into the bdelloplast milieu. To demonstrate that mCherry itself is not able to specifically localize outside the cell, we constructed a control strain expressing cytoplasmic mCherry under the control of the *bd3507* promoter. As expected, here mCherry fluorescence was detected only within B. bacteriovorus growing cells (Fig. 3A). Thus, it can be concluded that during host-dependent growth, Bd0934 and Bd3507 are specifically secreted into the surrounding bdelloplast.

We speculated that, during host-independent growth, these two nucleases are secreted into the medium. Thus, the host-independent derivatives of the strains that were used for fluorescence microscopy were isolated and grown in PY medium. The overnight cultures were separated into cell and supernatant fractions, and samples were subjected to SDS-PAGE followed by immunoblot detection of mCherry. Bd0934-mCherry and Bd3507-mCherry were found in the supernatant fraction, while cytoplasmic mCherry was detected in the cell fraction (Fig. 3B). Thus, immunoblot data strengthen and complement the fluorescence microscopy observations, and it is concluded that Bd0934 and Bd3507 are secreted extracellularly.

### The Δ*bd0934 Δbd3507* host-dependent strain exhibits higher extracellular DNase activity.

**FIG 4:**
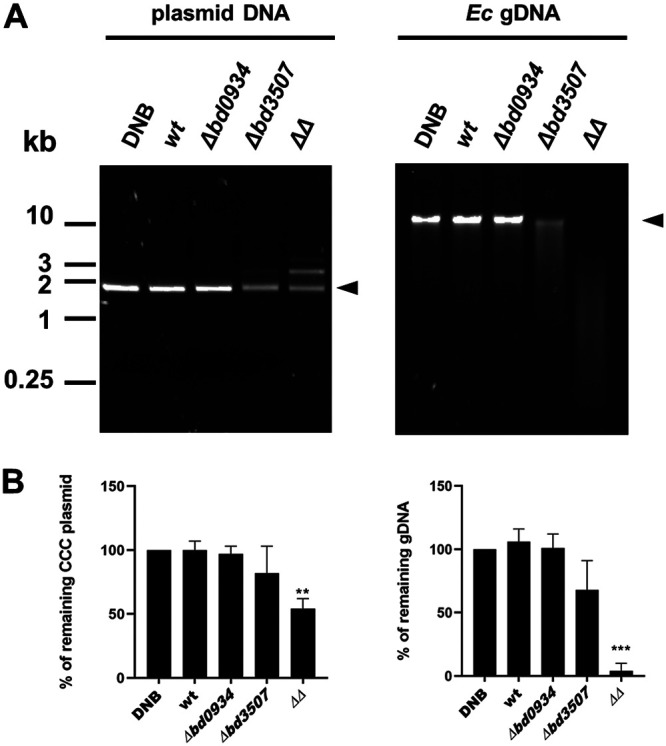
DNase assay demonstrating elevated extracellular nuclease activity in the supernatant of the Δ*bd0934* Δ*bd3507* strain (LHD110). Plasmid pUC19 (2.7 kb) (40 ng/μl) (left) or E. coli genomic DNA (40 ng/μl) (right) was mixed with an equal volume of supernatant collected from coculture of the indicated B. bacteriovorus strain (wt, strain HD100; Δ*bd0934*, strain LHD104; Δ*bd3507*, strain LHD103; ΔΔ [i.e., Δ*bd0934* Δ*bd3507*], strain LHD110). pUC19 and E. coli gDNA incubated with DNB medium served as negative controls. After 1 h of incubation at 37°C, samples were analyzed by 1% agarose gel electrophoresis. (A) A representative gel electrophoresis of three independent experiments. (B) Quantification of the remaining substrate, i.e., covalently closed circular (CCC) plasmid DNA or gDNA (bands indicated by arrowheads in panel A) relative to the control sample (DNB). Bars represent the average values ± SD from three independent DNase assays. A Student's *t* test determined statistical significance with respect to the wild-type strain (**, *P* ≤ 0.01; ***, *P* ≤ 0.001).

B. bacteriovorus extracellular DNase activity has been reported in several previously published studies (9, 14). To determine whether deletion of Bd0934 and/or Bd3507 affects this activity, a DNase assay was carried out using culture supernatant from the predatory host-dependent culture of the wild type and the respective deletion strains. Both plasmid DNA and E. coli genomic DNA (gDNA) were used as substrates (Fig. 4). In our hands, the extracellular DNase activity of the wild-type strain was almost undetectable (i.e., a weak activity was detected if the supernatant was concentrated [data not shown]). However, to our surprise, the double-deletion mutant strain exhibited higher DNase activity than the wild-type and single-deletion strains. The Δ*bd3507* strain also showed somewhat elevated DNase activity, while the activity in the Δ*bd0934* supernatant resembled that of the wild type. Such a pattern was observed in several repeated experiments. Although both substrates were digested by the supernatant of Δ*bd0934* Δ*bd3507* and Δ*bd3507* strains, the gDNA seems to be digested more efficiently than the plasmid DNA. This is clearly visible in the double-deletion strain, where all the substrate gDNA is digested into shorter fragments (visible as a smear), while the plasmid DNA is only partially digested to linear and nicked forms (the middle and upper band, respectively).

We were curious whether a similar trend would be apparent in the supernatant of host-independent cultures. As can be seen in Fig. S2A, all tested host-independent isolates displayed strong extracellular DNase activity, i.e., the substrate (gDNA) was fully degraded. The complete degradation of the substrate DNA made it difficult to conclude whether any of the supernatants had elevated nuclease activity. Thus, to avoid complete DNA degradation, we repeated the assay using a higher gDNA concentration, diluted supernatant, and shorter incubation time. We did not observe differences in nuclease activity between tested strains (Fig. S2B). Therefore, we conclude that the extracellular DNase activity of host-independent isolates is not noticeably affected by the deletion of *bd0934* and/or *bd3507*.

### Gene expression analysis of putative secreted nucleases.

**FIG 5:**
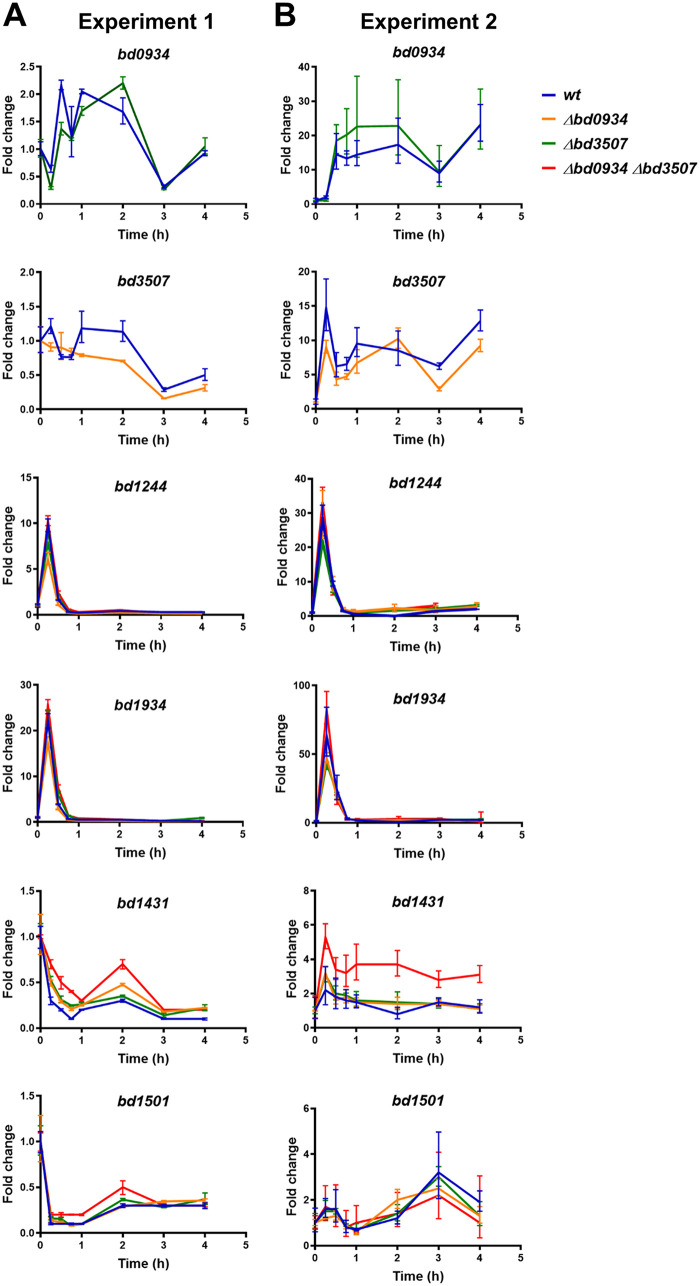
Temporal expression of genes encoding predicted nucleases: *bd0934*, *bd3507*, *bd1244*, *bd1431*, *bd1934,* and *bd1501*. Analysis was carried out for four B. bacteriovorus host-dependent strains: wt (HD100), Δ*bd0934* (LHD104), Δ*bd3507* (LHD103), and Δ*bd0934* Δ*bd3507* (LHD110) strains. RNA was isolated from samples collected at the indicated time points of the predatory cycle. Data obtained from qRT-PCR experiments were analyzed using a 2^−ΔΔ*CT*^ method. Experiments were repeated 2 times, and the results of each experiment are shown in separate panels (panel A, experiment 1; panel B, experiment 2). The MOI determined retrospectively was 2 for experiment 1 and 1 for experiment 2. Error bars represent standard deviations from a duplicate sample.

The observed higher nuclease activity in the double-deletion strain was unexpected but suggested that in the absence of both Bd0934 and Bd3507, some other nucleases are upregulated to compensate for this deficiency. To test this possibility, we compared the gene expression profiles of nucleases predicted to contain the signal peptide for secretion (*bd1244*, *bd1431*, *bd1934*, and *bd1501*) (Table S2) in the wild type and the double-deletion strain. Since the Δ*bd3507* strain showed a slightly elevated nuclease activity, we decided to also include single-deletion strains in the gene expression analysis (here, we additionally determined profiles for *bd0934* and *bd3507* in the respective deletion strain versus the wild type). Figure 5 summarizes the results of two independent experiments, in which samples for qRT-PCR analysis were collected from semisynchronous cultures throughout the whole B. bacteriovorus predatory life cycle. Both experiments yielded similar expression profiles of the analyzed nucleases. However, relative fold change values were generally higher in the second experiment than the first (Fig. 5). This difference is most likely a result of different multiplicity of infection (MOI) values for both experiments. That is, the reference gene (*dnaK*) expression level is constant in all B. bacteriovorus cells, while the expression of our gene of interest changes only in the cells that entered the growth phase inside the prey. Hence, the lower the MOI, the higher the relative fold change values for the gene of interest. In our analysis, we focused on the general trend of gene expression rather than on the fold change values.

The quantitative gene expression data for *bd0934* and *bd3507* in B. bacteriovorus HD100 (Fig. 5) are consistent with the semiquantitative analysis presented in Fig. 2. Both nucleases are upregulated during intracellular growth, with a broad peak of expression during the 30-min to 3-h time period for *bd0934* and the 1- to 2-h time period for *bd3507*. It seems that *bd3507* is also briefly upregulated at 15 min. Based on these results, we would expect the same temporal upregulation of the putative compensating nucleases. From Fig. 5, it is evident that the expression profile of *bd1244* and *bd1934* remains unchanged in the deletion mutant strain background, and both nucleases act only early during invasion of the prey cell (distinct single peak at 15 min). For *bd1431* and *bd1501*, the two independent experiments yielded slightly different expression profiles (see especially the time period of 0 to 1 h, all strains); therefore, these results are less conclusive. This apparent discrepancy can be explained by generally low relative expression levels of *bd1431* and *bd1501* throughout the predatory life cycle, together with the different MOI values for both experiments, as mentioned above. Nevertheless, it seems that *bd1501* might have a role in the late stages of intracellular growth, as the amount of transcript is increasing during the time period of 2 to 4 h. This observation is consistent in all analyzed strains. Between 1 h and 4 h, the level of *bd1431* transcript seems to be rather constant in the wild-type strain. However, it is notable that in both independent experiments the relative level of *bd1431* transcript is higher throughout the life cycle in the Δ*bd0934* Δ*bd3507* double deletion strain than in the wild-type strain. Expression profiles of all analyzed genes in the single-deletion strains resembled the wild-type expression profiles. Thus, the gene expression patterns over time were generally unchanged in the deletion mutant strains.

To enable better comparison of transcript levels at the specific time points, we have normalized the data relative to the wild-type strain and plotted the results of both experiments in a single graph (Fig. S3). We found indications of the downregulation of *bd0934* in the Δ*bd3507* background during early infection and significant downregulation of *bd3507* in the Δ*bd0934* background in the later stages of infection. Thus, it seems the deletion of one of these genes partly affects the expression of the other one. On the contrary, *bd1244*, *bd1431*, *bd1934,* and *bd1501* show trends of a slight increase in expression level in the Δ*bd0934* Δ*bd3507* background (at various time points of infection). Based on this observation, it can be speculated that the higher DNase activity seen in the double-deletion strain could be a result of generally increased transcription of other genes encoding secreted nucleases.

In summary, the qRT-PCR analysis did not pinpoint any specific nuclease that is strongly upregulated in the absence of Bd0934 and/or Bd3507, but slightly elevated transcript levels of *bd1431*, *bd1244*, *bd1934* and *bd1501* are observed in the Δ*bd0934* Δ*bd3507* background. Additionally, the detailed temporal analysis of gene expression revealed that during predatory growth, nucleases are expressed in a sequential manner (Fig. 5). An early brief expression of *bd1244* and *bd1934* is followed by expression of *bd0934* and *bd3507* in the midstages of development. The expression of *bd1501* seems to rise during the late stage of the predatory cycle.

### Expression of putative secreted nucleases in host-independent isolates.

**FIG 6:**
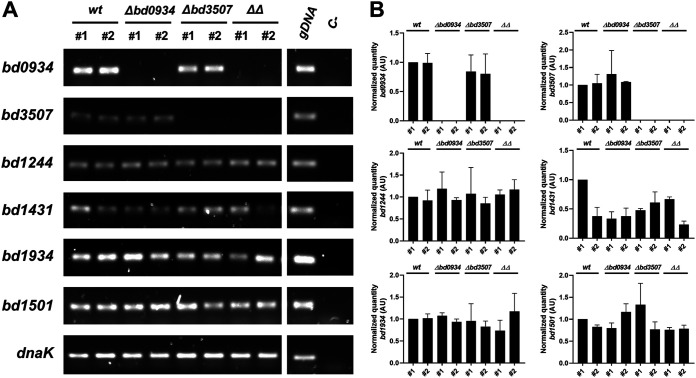
(A) Semi-qRT-PCR analysis to assess expression of *bd0934*, *bd3507*, *bd1244*, *bd1431*, *bd1934,* and *bd1501* in host-independent strains. Analysis was carried out for four B. bacteriovorus host-independent strains: LHI100 (wt), LHI106 (Δ*bd0934*), LHI105 (Δ*bd3507*), and LHI112 (ΔΔ, i.e., Δ*bd0934* Δ*bd3507*). Two independent isolates of each strain (labeled #1 and #2) were tested. The RNA used as a template in RT-PCR (10 ng) was isolated from samples of host-independent cultures grown in PY broth amended with 3 mM MgCl_2_ and 2 mM CaCl_2_. Equal volumes of each RT-PCR sample were analyzed by 1.5% agarose gel electrophoresis. *dnaK* is constitutively expressed in all strains and, thus, serves as an internal reference gene. gDNA, sample with genomic DNA from B. bacteriovorus used as a template (positive control); C-, sample with no template (negative control). A representative gel electrophoresis of two independent experiments is shown. (B) Intensity of bands for each gene was quantified relative to the sample wt #1 and normalized to intensity of the respective *dnaK* band. The average values ± SD from two independent semi-qRT-PCR experiments are plotted.

To get a full picture of the gene expression of selected nucleases, we have also analyzed samples collected from the host-independent isolates of the wild-type and nuclease-deficient strains. The rationale behind this analysis is the fact that *bd0934* and *bd3507* are also expressed during axenic growth, and no phenotype was associated with the deletion of both nucleases in the host-independent isolates. Thus, there was the possibility of a putative upregulation of compensating nucleases. Figure 6 shows no obvious upregulation of any nuclease in the deletion mutant background strains. With the exception of *bd1431*, all nucleases show similar expression levels in all tested strains. A dissimilar amount of *bd1431* transcript is observed in two isolates of the wild-type strain, suggesting that expression of this gene is variable in host-independent cells. Thus, no conclusion could be drawn on the potential upregulation of *bd1431* in the Bd0934- and Bd3507-deficient strains.

### Removal of E. coli biofilm and self-biofilm formation by nuclease-deficient strains.

B. bacteriovorus is known to efficiently degrade biofilms formed by various bacterial species, including some Gram-positive bacteria (14, 15). Extracellular DNA is one of the major components in many biofilms (16), and bacterial secreted DNases are sometimes involved in biofilm formation/eradication (12, 17–19). Thus, a biofilm removal assay was carried out to assess the ability of the nuclease-deficient strains to eradicate preformed prey biofilm. As can be concluded from Fig. S4, there is no difference in efficiency of biofilm removal between all tested strains. We then decided to analyze self-biofilm formation by host-independent derivatives of wild-type and nuclease-deficient strains. We observed a large variation in the ability to develop biofilm between individual isolates of each tested strain (Fig. S5). No consistent effect of nuclease deletion on self-biofilm formation was observed. Thus, we conclude that Bd0934 and Bd3507 are not essential for prey biofilm degradation or for self-biofilm formation.

## DISCUSSION

The unique lifestyle of bacterial predators requires the massive production of enzymes that function in the digestion of the prey’s macromolecules (6, 7). Amino acids obtained via degradation of host proteins constitute the main energy source for B. bacteriovorus growth, while nucleotides originating from host DNA and RNA serve as precursors for the synthesis of the predator nucleic acids (3). Although proteomic and transcriptomic studies provided some insight into the B. bacteriovorus HD100 predatosome (a group of unique proteins involved in prey killing and digestion) (6, 20, 21), the exact role of the majority of such enzymes has yet to be elucidated.

Data presented in our paper suggest that Bd0934 and Bd3507 are also part of the predatosome. A detailed temporal analysis of transcript levels revealed that these two putative DNases are upregulated during the midstages of intracellular growth. Such complete gene expression profiles complement the data available from previous large transcriptomic studies, where only one or two time points of the predatory cycle were selected to determine the predatosome (6, 20). In the microarray study by Lambert et al. (6), *bd0934* and *bd3507* were classified as genes specific for host-independent growth due to their significant upregulation in the host-independent (HI) cells versus attack phase cells. In the same study, no significant upregulation was observed in the cells during the early phase of predation (i.e., at 30 min postinfection) versus attack phase cells. Our results are partly in line with these observations, as levels of *bd0934* and *bd3507* transcripts in the HI cells is indeed higher than that in cells at 0 h in predatory culture (Fig. 2). However, contrary to observations by Lambert et al., we do see the upregulation of *bd0934* and *bd3507* at 30 min into the predatory cycle (Fig. 2 and 5). A possible explanation of these differences is that Lambert et al. used pure attack phase cells (without added prey) as a reference sample, while in our study the reference is the sample collected from the predatory culture at time point 0 h (and, thus, consists of both the prey and the predator, leading to possible immediate effects). Regardless of the discrepancy concerning expression at the early stages of predation, our data show that both genes are certainly expressed during midstages of intracellular growth. This was also proved in the transcriptome sequencing study by Karunker et al. (20), which showed high abundance of *bd0934* and *bd3507* transcript in the cells collected at 180 min postinfection. Additionally, our fluorescence microscopy data demonstrated that Bd0934 and Bd3507 are secreted into the bdelloplast milieu during predatory growth. Taken together, these findings suggest the involvement of Bd0934 and Bd3507 in the degradation of the host DNA. It is interesting, though, that both nucleases can be deleted without any effect on predation. Considering the complexity of the predatory cycle and its importance for B. bacteriovorus survival, the high enzyme redundancy should not be surprising. Lack of Bd0934 and Bd3507 may be compensated by another nuclease(s) with the same activity and extracellular localization (as discussed below). A lack of phenotype concerning predation ability was also observed in the Δ*bd1244* Δ*bd1934* double-deletion mutant strain constructed by Lambert and Sockett (12). Bd1244 and Bd1934 are supposedly acting in the early stage of predation (as also confirmed by our qRT-PCR analysis), but the double-deletion mutant showed no difference in rate of predation compared to that of the wild type. Surprisingly, the Δ*bd1244* Δ*bd1934* strain was less efficient in self-biofilm formation and performed better in clearance of preformed prey biofilm than the wild-type strain. Based on these previous findings, we carried out analogous biofilm analysis of our mutant strains. We did not find any differences in biofilm formation/removal between all tested strains, which implies no involvement (or redundancy) of Bd0934 and Bd3507 in these processes.

Deletion of *bd0934* and *bd3507* led to elevated extracellular DNase activity, a phenotype that is contrary to what would be intuitively expected. It implies that some response mechanism is triggered in the cells to compensate for Bd0934 and Bd3507 deficiency. One possible response is the upregulation of another DNase(s) with the corresponding subcellular localization and specificity. Our qRT-PCR analysis, which covered nucleases with predicted signal peptide, suggests that it could be an overall slight increase in expression of the remaining nucleases (especially the nuclease encoded by *bd1431*). Upregulation of another, unidentified extracellular nuclease(s) is also possible. It cannot be ruled out that the elevated DNase activity is a result of a mechanism other than transcriptional regulation. As proven in multiple studies, mRNA expression levels do not always reflect the protein abundance in the cell (22). Increased translation rate of compensating nuclease (coupled to its elevated secretion) can be the reason for the observed higher DNase activity. Finally, changes in enzyme activity can hypothetically explain the observed phenotype, i.e., it is possible that in the presence of Bd0934 and Bd3507, the activity of another extracellular nuclease is quenched, and such an enzyme becomes activated in the double-deletion strain. The main finding of our study is the observed sequential production of putative extracellular nucleases during the predatory cycle. This observation complements the model suggested by Rittenberg and coworkers (9, 11). Their studies demonstrated that within the first 45 to 60 min of the predatory cycle, the host genomic DNA is digested into intermediate-size fragments of approximately 780 bp on average. Subsequently, the synthesis of B. bacteriovorus DNA begins and seems to occur at a rate similar to that of the breakdown of host DNA fragments into single nucleotides. Thus, the released nucleotide monomers appear to be immediately incorporated into the newly synthesized genome of the predator. Such controlled digestion of the host nucleic acid was suggested to be a result of the continuous and sequential synthesis of *Bdellovibrio* DNases with different specificities and activities. Thus, the following scenario can be suggested based on our qRT-PCR data and the kinetic studies of host DNA degradation by Rosson and Rittenberg. Bd1244 and Bd1934 (and possibly Bd3507) could be the first acting enzymes responsible for rapid endonucleolytic attack on the host DNA. The resulting DNA fragments subsequently could be broken down into shorter oligomers by the action of Bd0934 and Bd3507. As concluded by Rosson and Rittenberg, the subsequent digestion into nucleotide monomers is catalyzed by exonucleases that are synthesized approximately 30 min into the predatory cycle and continue to act throughout the *Bdellovibrio* growth phase. *bd0934* and *bd3507* are continuously expressed after 30 min into the predatory cycle, and their initial expression (especially *bd0934*) is timed to the initiation of *Bdellovibrio* DNA synthesis (Fig. S6). However, both Bd0934 and Bd3507 are predicted to be endonucleases and, as such, do not match the model suggested by Rosson and Rittenberg. The determination of their nuclease specificity *in vitro* could be helpful to understand their role in the degradation of the host DNA. Likewise, detailed *in vivo* studies on Bd1501 and Bd1431 are needed to establish whether they are part of the predatosome or not.

## MATERIALS AND METHODS

### Bacterial strains and growth media.

Bacterial strains used in this work are listed in Table 1. E. coli TOP10 was used for plasmid DNA propagation, E. coli DH5α was used for prey in predatory cocultures, and E. coli S17 served as a donor strain in conjugation experiments and was used for the development of prey biofilm. E. coli strains were grown at 37°C in YT broth (23) or on LB agar plates (24).

B. bacteriovorus strains were routinely grown at 29°C, 200 rpm, in DNB medium (0.08% nutrient broth, 0.05% Casamino Acids, 0.01% yeast extract [pH 7.6], supplemented with 3 mM CaCl_2_ and 2 mM MgCl_2_ after autoclaving) (25) with E. coli DH5 as the host cell (8 ml DNB, 0.45 ml of E. coli DH5α overnight culture, 0.15 ml of 7- to 10-day-old B. bacteriovorus lysate). Overnight *Bdellovibrio* lysates were passed through 0.45-μm filters. In semisynchronous growth experiments, B. bacteriovorus strains were grown in HM buffer (25 mM HEPES [pH 7.6] supplemented with 3 mM CaCl_2_ and 2 mM MgCl_2_) (26). Reviving B. bacteriovorus strains from frozen stock and plaque-forming unit (PFU) enumeration was done by an overlay agar technique using YPSC medium (0.05% yeast extract, 0.05% peptone, 0.025% CH_3_COONa, 0.0125% MgSO_4_ [pH 7.6], with CaCl_2_ added after autoclaving to give a concentration of 0.025%), as described by Lambert and Sockett (23). Host-independent (HI) strains were grown in PY medium (1% peptone, 0.3% yeast extract [pH 6.8]) amended with 3 mM CaCl_2_ and 2 mM MgCl_2_ as described in Lambert and Sockett (23).

When needed, media were supplemented with antibiotics at the following concentrations: ampicillin at 100 μg ml^−1^, kanamycin at 40 μg ml^−1^ and chloramphenicol at 12.5 μg ml^−1^.

### DNA techniques and plasmid construction.

DNA manipulation was performed by standard methods (24). Plasmid DNA and genomic DNA were isolated using the GeneJET plasmid miniprep kit (Thermo Scientific) and the GenElute bacterial genomic DNA kit (Sigma), respectively. PCR was carried out using Phusion high-fidelity DNA polymerase (New England Biolabs). The sequences of primers used in PCR are listed in Table S1 in the supplemental material. The synthetic DNA fragments were purchased from GenScript, USA. DNA ligation was performed using T4 DNA ligase (New England Biolabs). One-shot TOP10 chemically competent E. coli cells (Invitrogen) were used for transformation. Plasmids used in this study are listed in Table 1. All DNA fragments cloned into plasmids were verified by sequencing.

**(i) Construction of pEBF1.** A 2,240-bp-long DNA fragment, consisting of the 700-bp region upstream of *bd0934*, chloramphenicol cassette, and the 700-bp region downstream of *bd0934* was synthesized. The fragment was flanked by BamHI and HindIII restriction sites at the 5′ and 3′ end, respectively. The fragment was cloned into pK18mobsacB using BamHI and HindIII restriction sites, resulting in plasmid pEBF1.

**(ii) Construction of pEBF2.** pEBF2 was constructed in the same way as pEBF1, but the synthetic DNA fragment (2,240 bp) comprised a 700-bp region upstream of *bd3507*, a chloramphenicol cassette, and a 700-bp region downstream of *bd3507*. The DNA fragment was cloned into pK18mobsacB using the flanking restriction sites, i.e., BamHI and SphI (at the 5′ and 3′ end of the insert, respectively).

**(iii) Construction of pEBF5.** An 810-bp-long fragment, comprising a chloramphenicol cassette, was cut out from pEBF2 using SalI restriction sites. The remaining linear DNA fragment of pEBF2 (7,119 bp) was circularized by ligation, yielding plasmid pEBF5, which contains a 700-bp region upstream of *bd3507* and a 700-bp region downstream of *bd3507*.

**(iv) Construction of pEBF6.** A 1,674-bp DNA fragment was synthesized, comprising the promoter region and the coding sequence of *bd0934* (without the stop codon), the linker sequence (encoding LEVDGIDKLDDP), and the sequence encoding mCherry (in-frame with the *bd0934* coding sequence and the linker). The synthetic fragment was flanked by HindIII and BamHI restriction sites (at the 5′ and 3′ end, respectively), and these sites were used to clone it into pPROBE-NT. The resulting plasmid was named pEBF6.

**(v) Construction of pEBF7.** pEBF7 was constructed analogously to pEBF6, but the synthetic DNA fragment (2,008 bp) comprised a sequence encoding the translational fusion of Bd3507 to mCherry under the native *bd3507* promoter.

**(vi) Construction of pEBF11.** pEBF11 was constructed by a site-directed mutagenesis approach (Phusion site-directed mutagenesis; Thermo Scientific) using primers E038 and E040 (both phosphorylated at the 5′ end) and plasmid pEBF7 as the template. The resulting PCR product (7,882 bp) was circularized by ligation, resulting in plasmid pEBF11. It contains the sequence comprised of the *bd3507* promoter, the start codon of *bd3507*, and the sequence encoding mCherry (in-frame with the start codon of *bd3507*).

### Construction of B. bacteriovorus strains.

All B. bacteriovorus strains described in this work are derivatives of strain HD100 (Table 1). Deletion of *bd0934* and *bd3507* was carried out as described in Steyert and Pineiro (27), with modifications. Briefly, the suicide plasmid pEBF1 was transferred to B. bacteriovorus HD100 via conjugal mating with E. coli S17/pEBF1 as described by Cotter and Thomashow (28). Successful conjugants were selected via plating on YPSC overlay plates supplemented with kanamycin and containing E. coli TOP10/pFW13 as the prey in the top agar layer. Plaques that appeared after several days of incubation at 29°C were tested by PCR for integration of the entire plasmid at the flanking region of *bd0934* by a single-crossover event. The resulting merodiploid strain (LHD101) was then grown for 24 h in liquid coculture in DNB medium (without antibiotic selection) to allow excision of the plasmid by a second crossover event. Subsequently, an aliquot of this coculture was transferred to a suspension of E. coli DH5α in HM buffer (optical density at 600 nm [OD_600_] of 1) supplemented with 5% sucrose for selection of excisants. The coculture was incubated at 29°C, 200 rpm, until lysis of prey was visible (approximately 48 h). Serial dilutions of the lysed coculture were plated on YPSC overlay plates supplemented with chloramphenicol and containing E. coli TOP10/pDC123 as the prey. Plaques that appeared after several days of incubation at 29°C were tested by PCR for a successful second recombination event, which resulted in the replacement of *bd0934* coding sequence by a chloramphenicol cassette. The constructed strain was named LHD104. Strain LHD103, in which *bd3507* was replaced by a chloramphenicol cassette, was constructed essentially as described for LHD104, i.e., plasmid pEBF2 was conjugally transferred into B. bacteriovorus HD100, yielding merodiploid strain LHD102. Subsequent counterselection with sucrose and selection for chloramphenicol resistance yielded strain LHD103. The double-deletion mutant strain, LHD110, was constructed by conjugal transfer of pEBF5 into strain LHD104. The resulting merodiploid strain LHD107 was subjected to counterselection on sucrose. Since plasmid pEBF5 carries an in-frame markerless deletion of *bd3507*, no antibiotic selection was used after growth in the presence of sucrose. Successful excisant with an in-frame markerless deletion of *bd3507* was detected by PCR, and the strain was named LHD110.

Plasmid pEBF6 was conjugally transferred into strain LHD104, yielding strain LHD104/pEBF6. Plasmids pEBF7 and pEBF11 were conjugally transferred into strain LHD103, yielding strains LHD103/pEBF7 and LHD103/pEBF11, respectively.

All host-independent B. bacteriovorus strains were generated by plating 0.45-μm-filtered lysates of host-dependent strains on PY agar plates as described in Lambert and Sockett (23).

### DNase activity assay.

Twenty microliters of plasmid pUC19 (40 ng/μl), or E. coli genomic DNA (40 ng/μl), was mixed with an equal volume of B. bacteriovorus supernatant collected from 16-h-old DNB coculture (HD strains) or 11- to 24-h-old culture of HI strains grown in PY medium supplemented with 3 mM CaCl_2_ and 2 mM MgCl_2_. Reaction mixtures were incubated at 37°C for 1 h. Nucleolytic degradation of DNA was analyzed by agarose gel electrophoresis.

### Semi-qRT-PCR and qRT-PCR.

Semisynchronous cocultures of B. bacteriovorus were set as described in the “Predatory kill curves and growth curves” section of the supplemental material. Samples for isolation of RNA were taken at the following time points of the predatory cycle: 0 min, 15 min, 30 min, 45 min, 1 h, 2 h, 3 h, and 4 h. HI strains were grown as described in the supplemental material, and samples were collected from the exponentially growing cultures (OD_600_ of ∼0.6). All samples were immediately mixed with the RNAprotect bacterial reagent (Qiagen). RNA was purified using the RNeasy minikit (Qiagen), including an on-column DNase treatment step during the RNA isolation procedure. Despite the DNase treatment, traces of DNA were detectable in the isolated RNA (as determined by PCR). Thus, an extra DNase treatment (in solution) was required and RNA was cleaned up using the RNeasy minikit (Qiagen) according to the manufacturer’s guidelines. Ten nanograms of the isolated RNA was used as a template in PCRs. Reverse transcription and PCR were performed in one step using the Power SYBR green RNA-to-CT one-step kit (Applied Biosystems). qRT-PCR was performed according to the manufacturer’s protocol. In semi-qRT-PCR, the number of cycles was reduced to 25, and the resulting PCR products were analyzed by agarose gel electrophoresis. Primer pairs used for the detection of *bd0934*, *bd1244*, *bd1431*, *bd1501*, *bd1934*, *bd3507,* and *dnaK* are listed in Table S1. Appropriate negative-control reactions were carried out: with no template, no RT enzyme mix, and genomic DNA from E. coli as a template. Real-time PCR amplification was carried out in a QuantStudio 7 Flex real-time PCR system (Applied Biosystems). The relative expression of genes of interest (*bd0934*, *bd1244*, *bd1431*, *bd1501*, *bd1934*, and *bd3507*) was calculated using the 2^−ΔΔCT^ method (29). The *dnaK* gene was chosen as an internal control gene (30–33), and the sample collected at 0 min was used as a calibrator. All experiments were repeated at least 2 times.

### Epifluorescence and DIC microscopy.

Semisynchronous cocultures of B. bacteriovorus were set as described in the supplemental material, with the exception that HM buffer was supplemented with kanamycin and E. coli TOP10/pFW13 was used as the prey. Five microliters of the culture was taken out at the indicated time point and mounted on an agarose-coated microscopy slide. Differential interference contrast (DIC) and fluorescence images were acquired using a Nikon Ti Eclipse microscope equipped with a Spectra X light source (Lumencor) and an Ixon Ultra DU897 electron-multiplying charge-coupled device camera (Andor). Exposure time for mCherry was 2 s. Images were processed in NIS-Elements v4.51 and saved in TIFF format.

### Western blotting.

B. bacteriovorus HI strains were grown as described in the supplemental material, with the exception that PY medium was supplemented with kanamycin. Chymostatin (1×) (protease inhibitor set; G‐Biosciences, USA) was added to the cultures to prevent proteolytic degradation of the fusion protein. After 24 h of incubation, the optical density was measured and adjusted for all tested strains to the value of 0.4. Subsequently, a sample of 180 μl was taken from each culture and centrifuged at 10,000 × *g* for 10 min at room temperature. Supernatant was transferred to a fresh tube and saved for further analysis. The remaining pellet was resuspended in the initial volume of fresh PY medium, and cells were sonicated (5 times for 30 s, with 30-s breaks between cycles). Equal volumes of both fractions, the supernatant and the pellet, were loaded on 4 to 15% Mini-PROTEAN TGX precast protein gels (Bio-Rad). After SDS-PAGE was completed, the proteins were transferred onto a polyvinylidene difluoride (PVDF) membrane using the Trans-Blot Turbo system (Bio-Rad). Immunodetection was carried out using polyclonal anti-mCherry primary antibodies from rabbit (1:1,000; Thermo Scientific) and anti-rabbit horseradish peroxidase (HRP)-conjugated secondary antibodies from donkey (1:5,000; Jackson ImmunoResearch Laboratories). Clarity Western ECL substrate (Bio-Rad) was used as a substrate for HRP, and luminescence was detected using a GelDoc imager (Bio-Rad).

## Supplementary Material

Supplemental file 1

## References

[1] Dashiff A, Junka RA, Libera M, Kadouri DE. 2011. Predation of human pathogens by the predatory bacteria *Micavibrio aeruginosavorus* and *Bdellovibrio bacteriovorus*. J Appl Microbiol 110:431–444. doi:10.1111/j.1365-2672.2010.04900.x. 21114596

[2] Kadouri DE, To K, Shanks RMQ, Doi Y. 2013. Predatory bacteria: a potential ally against multidrug-resistant Gram-negative pathogens. PLoS One 8:e63397. doi:10.1371/journal.pone.0063397. 23650563 PMC3641118

[3] Rotem O, Pasternak Z, Jurkevitch E. 2014. The genus Bdellovibrio and like organisms, p 3–17. *In* Rosenberg E, DeLong EF, Lory S, Stackenbrandt E, Thompson F (ed), The prokaryotes: Deltaproteobacteria and Epsilonproteobacteria. Springer, New York, NY. doi:10.1007/978-3-642-39044-9_379.

[4] Sockett RE. 2009. Predatory lifestyle of *Bdellovibrio bacteriovorus*. Annu Rev Microbiol 63:523–539. doi:10.1146/annurev.micro.091208.073346. 19575566

[5] Rotem O, Pasternak Z, Shimoni E, Belausov E, Porat Z, Pietrokovski S, Jurkevitch E. 2015. Cell-cycle progress in obligate predatory bacteria is dependent upon sequential sensing of prey recognition and prey quality cues. Proc Natl Acad Sci U S A 112:E6028–E6037. doi:10.1073/pnas.1515749112. 26487679 PMC4640792

[6] Lambert C, Chang C-Y, Capeness MJ, Sockett RE. 2010. The first bite—profiling the predatosome in the bacterial pathogen *Bdellovibrio*. PLoS One 5:e8599. doi:10.1371/journal.pone.0008599. 20062540 PMC2797640

[7] Rendulic S, Jagtap P, Rosinus A, Eppinger M, Baar C, Lanz C, Keller H, Lambert C, Evans KJ, Goesmann A, Meyer F, Sockett RE, Schuster SC. 2004. A predator unmasked: life cycle of *Bdellovibrio bacteriovorus* from a genomic perspective. Science 303:689–692. doi:10.1126/science.1093027. 14752164

[8] Seidler RJ, Starr MP. 1969. Isolation and characterization of host-independent Bdellovibrios. J Bacteriol 100:769–785. doi:10.1128/JB.100.2.769-785.1969. 4901359 PMC250157

[9] Rosson RA, Rittenberg SC. 1979. Regulated breakdown of *Escherichia coli* deoxyribonucleic acid during intraperiplasmic growth of *Bdellovibrio bacteriovorus* 109J. J Bacteriol 140:620–633. doi:10.1128/JB.140.2.620-633.1979. 387743 PMC216690

[10] Hespell RB, Miozzari GF, Rittenberg SC. 1975. Ribonucleic acid destruction and synthesis during intraperiplasmic growth of *Bdellovibrio bacteriovorus*. J Bacteriol 123:481–491. doi:10.1128/JB.123.2.481-491.1975. 1097411 PMC235752

[11] Matin A, Rittenberg SC. 1972. Kinetics of deoxyribonucleic acid destruction and synthesis during growth of *Bdellovibrio bacteriovorus* strain 109D on *Pseudomonas putida* and *Escherichia coli*. J Bacteriol 111:664–673. doi:10.1128/JB.111.3.664-673.1972. 4559819 PMC251338

[12] Lambert C, Sockett RE. 2013. Nucleases in *Bdellovibrio bacteriovorus* contribute towards efficient self-biofilm formation and eradication of preformed prey biofilms. FEMS Microbiol Lett 340:109–116. doi:10.1111/1574-6968.12075. 23297829 PMC3593177

[13] Barel G, Jurkevitch E. 2001. Analysis of phenotypic diversity among host-independent mutants of *Bdellovibrio bacteriovorus* 109J. Arch Microbiol 176:211–216. doi:10.1007/s002030100312. 11511869

[14] Monnappa AK, Dwidar M, Seo JK, Hur J-H, Mitchell RJ. 2014. *Bdellovibrio bacteriovorus* inhibits *Staphylococcus aureus* biofilm formation and invasion into human epithelial cells. Sci Rep 4:3811–3811. doi:10.1038/srep03811. 24448451 PMC3898049

[15] Kadouri D, O'Toole GA. 2005. Susceptibility of biofilms to *Bdellovibrio bacteriovorus* attack. Appl Environ Microbiol 71:4044–4051. doi:10.1128/AEM.71.7.4044-4051.2005. 16000819 PMC1169041

[16] Montanaro L, Poggi A, Visai L, Ravaioli S, Campoccia D, Speziale P, Arciola CR. 2011. Extracellular DNA in biofilms. Int J Artif Organs 34:824–831. doi:10.5301/ijao.5000051. 22094562

[17] Seper A, Fengler VHI, Roier S, Wolinski H, Kohlwein SD, Bishop AL, Camilli A, Reidl J, Schild S. 2011. Extracellular nucleases and extracellular DNA play important roles in *Vibrio cholerae* biofilm formation. Mol Microbiol 82:1015–1037. doi:10.1111/j.1365-2958.2011.07867.x. 22032623 PMC3212620

[18] Tan A, Li W-S, Verderosa AD, Blakeway LV, D Mubaiwa T, Totsika M, Seib KL. 2019. *Moraxella catarrhalis* NucM is an entry nuclease involved in extracellular DNA and RNA degradation, cell competence and biofilm scaffolding. Sci Rep 9:2579. doi:10.1038/s41598-019-39374-0. 30796312 PMC6384898

[19] Beenken KE, Spencer H, Griffin LM, Smeltzer MS. 2012. Impact of extracellular nuclease production on the biofilm phenotype of Staphylococcus aureus under *in vitro* and *in vivo* conditions. Infect Immun 80:1634–1638. doi:10.1128/IAI.06134-11. 22354028 PMC3347440

[20] Karunker I, Rotem O, Dori-Bachash M, Jurkevitch E, Sorek R. 2013. A global transcriptional switch between the attack and growth forms of *Bdellovibrio bacteriovorus*. PLoS One 8:e61850. doi:10.1371/journal.pone.0061850. 23613952 PMC3627812

[21] Dori-Bachash M, Dassa B, Pietrokovski S, Jurkevitch E. 2008. Proteome-based comparative analyses of growth stages reveal new cell cycle-dependent functions in the predatory bacterium *Bdellovibrio bacteriovorus*. Appl Environ Microbiol 74:7152–7162. doi:10.1128/AEM.01736-08. 18836011 PMC2592910

[22] Liu Y, Beyer A, Aebersold R. 2016. On the dependency of cellular protein levels on mRNA abundance. Cell 165:535–550. doi:10.1016/j.cell.2016.03.014. 27104977

[23] Lambert C, Sockett RE. 2008. Laboratory maintenance of *Bdellovibrio*. Curr Protoc Microbiol Chapter 7:Unit 7B.2. doi:10.1002/9780471729259.mc07b02s9. 18770540

[24] Sambrook J, Russell D. 2001. Molecular cloning: a laboratory manual, vol 1, 3rd ed. Cold Spring Harbor Laboratory Press, Cold Spring Harbor, NY.

[25] Ruby EG. 1992. The genus *Bdellovibrio*, p 3400–3415. *In* Balows A, Trüper HG, Dworkin M, Harder W, Schleifer K-H (ed), The prokaryotes: a handbook on the biology of bacteria: ecophysiology, isolation, identification, applications. Springer New York, New York, NY.

[26] Jurkevitch E. 2006. Isolation and classification of *Bdellovibrio* and like organisms. Curr Protoc Microbiol Chapter 7:Unit 7B.1. doi:10.1002/9780471729259.mc07b01s00. 18770593

[27] Steyert SR, Pineiro SA. 2007. Development of a novel genetic system to create markerless deletion mutants of *Bdellovibrio bacteriovorus*. Appl Environ Microbiol 73:4717–4724. doi:10.1128/AEM.00640-07. 17557848 PMC1951038

[28] Cotter TW, Thomashow MF. 1992. A conjugation procedure for *Bdellovibrio bacteriovorus* and its use to identify DNA sequences that enhance the plaque-forming ability of a spontaneous host-independent mutant. J Bacteriol 174:6011–6017. doi:10.1128/jb.174.19.6011-6017.1992. 1400153 PMC207665

[29] Livak KJ, Schmittgen TD. 2001. Analysis of relative gene expression data using real-time quantitative PCR and the 2^-ΔΔCT^ method. Methods 25:402–408. doi:10.1006/meth.2001.1262. 11846609

[30] Lowry RC, Milner DS, Al-Bayati AMS, Lambert C, Francis VI, Porter SL, Sockett RE. 2019. Evolutionary diversification of the RomR protein of the invasive deltaproteobacterium, *Bdellovibrio bacteriovorus*. Sci Rep 9:5007. doi:10.1038/s41598-019-41263-5. 30899045 PMC6428892

[31] Capeness MJ, Lambert C, Lovering AL, Till R, Uchida K, Chaudhuri R, Alderwick LJ, Lee DJ, Swarbreck D, Liddell S, Aizawa S-I, Sockett RE. 2013. Activity of *Bdellovibrio hit* locus proteins, Bd0108 and Bd0109, links type IVa pilus extrusion/retraction status to prey-independent growth signalling. PLoS One 8:e79759. doi:10.1371/journal.pone.0079759. 24224002 PMC3818213

[32] Kuru E, Lambert C, Rittichier J, Till R, Ducret A, Derouaux A, Gray J, Biboy J, Vollmer W, VanNieuwenhze M, Brun YV, Sockett RE. 2017. Fluorescent D-amino-acids reveal bi-cellular cell wall modifications important for *Bdellovibrio bacteriovorus* predation. Nat Microbiol 2:1648–1657. doi:10.1038/s41564-017-0029-y. 28974693 PMC5705579

[33] Cadby IT, Basford SM, Nottingham R, Meek R, Lowry R, Lambert C, Tridgett M, Till R, Ahmad R, Fung R, Hobley L, Hughes WS, Moynihan PJ, Sockett RE, Lovering AL. 2019. Nucleotide signaling pathway convergence in a cAMP-sensing bacterial c-di-GMP phosphodiesterase. EMBO J 38:e100772. doi:10.15252/embj.2018100772. 31355487 PMC6717892

[34] Podbielski A, Spellerberg B, Woischnik M, Pohl B, Lutticken R. 1996. Novel series of plasmid vectors for gene inactivation and expression analysis in group A streptococci (GAS). Gene 177:137–147. doi:10.1016/0378-1119(96)84178-3. 8921859

[35] Schäfer A, Tauch A, Jäger W, Kalinowski J, Thierbach G, Pühler A. 1994. Small mobilizable multi-purpose cloning vectors derived from the *Escherichia coli* plasmids pK18 and pK19: selection of defined deletions in the chromosome of *Corynebacterium glutamicum*. Gene 145:69–73. doi:10.1016/0378-1119(94)90324-7. 8045426

[36] Miller WG, Leveau JH, Lindow SE. 2000. Improved *gfp* and *inaZ* broad-host-range promoter-probe vectors. Mol Plant Microbe Interact 13:1243–1250. doi:10.1094/MPMI.2000.13.11.1243. 11059491

