# Supplementary material for: Studies on Bd0934 and Bd3507, Two Secreted Nucleases from Bdellovibrio bacteriovorus, Reveal Sequential Release of Nucleases during the Predatory Cycle
Source: J Bacteriol. 2020 Aug 25;202(18):e00150-20. doi: 10.1128/JB.00150-20 (PMC7925074; doi:10.1128/JB.00150-20)
Supplement: Supplemental file 1 [file JB.00150-20-s0001.pdf]

Supporting Information

Studies on Bd0934 and Bd3507, two secreted nucleases from *Bdellovibrio bacteriovorus*, reveal sequential release of nucleases during the predatory cycle.

Ewa Bukowska-Faniband, Tilde Andersson, Rolf Lood

Department of Clinical Sciences Lund, Division of Infection Medicine, Lund University, Sweden

Supplementary Material and Methods

Predatory kill curves and growth curves

Data for predatory kill curves and growth curves were acquired using *Bioscreen C* Automated Microbiology Growth Curve Analysis System (Growth Curves USA).

Semi-synchronous cultures of *B. bacteriovorus* host-dependent strains were prepared as follows. Fresh attack-phase bdellovibrio cells from 0.45 µm-filtered overnight DNB coculture were centrifuged at 5500g, 20 min, RT. The resulting pellet was resuspended in 1/10 of the initial volume of the HM buffer. The average pfu/ml of such concentrated *B. bacteriovorus* suspension was 2×10^9, as determined by the overlay agar technique. *E. coli* DH5α prey was grown in YT medium until the OD_{600} reached 0.8-1. Cells were spun down at 5000g, 10 min, washed with HM buffer and spun down again. Finally, the pellet was resuspended in HM buffer to an OD_{600}=1 and stored at 4°C until use (*i.e.* 1-2 days). The average cfu/ml of such prey suspension was 3×10^9, as determined by plating of serial dilutions on LB agar plates. *B.*

bacteriovorus was mixed with *E. coli* prey at the m.o.i. ~3 (unless stated otherwise) and 0.2 ml of such semi-synchronous predatory culture was transferred to a microplate well. Each strain was grown in triplicates. The following settings were used to acquire data for predatory kill curves: i) incubation temperature: 29°C, ii) shaking: medium, iii) wavelength: 600 nm. The reduction in OD (*i.e.* lysis of the prey) was measured every 15 minutes for 8 hours.

B. bacteriovorus HI strains were grown in PY medium amended with 3 mM $CaCl_2$ and 2 mM $MgCl_2$ as described in Lambert and Sockett (1). A 1 to 2-days-old culture (OD_{600}~1.5) was back-diluted into fresh medium to OD_{600}=0.1, and 0.2 ml was transferred to a microplate well for the growth curve experiment. Each culture was grown in five technical replicates. The instrument settings were the same as for predatory kill curves experiment, but the OD measurement was taken every 30 min, for 48h. Two independent isolates of each HI strain were tested.

Predatory kill curves and growth curves experiments were repeated at least 3 times.

Prey biofilm removal by HD strains

The prey biofilm removal assay was carried out as described by Lambert and Sockett (2) with modifications. Briefly, an *E. coli* S17 overnight culture grown in YT medium was diluted in fresh YT medium to OD=0.1, and 180 µl was dispensed into each well of a 96-well PVC plate (Corning). The plate was incubated at 29°C for 24h, in order to develop an *E. coli* prey biofilm. Following incubation, the remaining planktonic cells were washed off with DNB medium. Subsequently, 180 µl of fresh *Bdellovibrio* lysate (0.45 µm-filtered) obtained from the routine DNB coculture was added into each well with the preformed biofilm (in technical quadruplicates for each *B. bacteriovorus* strain tested). As a negative control, DNB medium was added instead of *B. bacteriovorus* lysate. The plate was incubated for another 24h at 29°C. *Bdellovibrio* cells were removed from wells by triple gentle washes with water and the

remaining biofilm was quantified by crystal violet staining as follows. The biofilm was fixed with 99% methanol for 15 minutes. After removal of methanol the plate was allowed to air dry for 10 minutes. Subsequently, the biofilm was stained with 0.1% crystal violet (w/v in water) for 4 minutes, followed by triple gentle washes with water. The dye was extracted with 20:80 acetone-ethanol (vol/vol) and transferred to a fresh microtiter plate for reading the absorbance at 600 nm. Student's *t*-test was performed on data sets to test statistical significance. The assay was repeated at least 3 times.

Biofilm formation by HI strains

Biofilm formation assays for *B. bacteriovorus* HI strains were carried out essentially as described by Lambert and Sockett (2), with exception that the crystal violet staining was performed as described above. Two independent isolates of each HI strain were tested (10 technical replicates per isolate). Student's *t*-test was performed on data sets to test statistical significance. The assay was repeated at least 3 times.

Table S1. Primers used in this work

Name	Sequence	Description
E038	5’ PHO-ATGGTGAGCAAGGGCGAGGA 3’	Primer used for site-directed mutagenesis
E040	5’ PHO-CACAAAGAACCTCCATAGTCAGGCA 3’	Primer used for site-directed mutagenesis
E027	5’ ACAGTCGCTCAATCCTGGTT 3’	qPCR primer; detection of *bd0934*
E028	5’ GCCCAGTTTGGATTCTGTGT 3’	qPCR primer; detection of *bd0934*
E029	5’ ACTTGTACCCGACCGACAAC 3’	qPCR primer; detection of *bd3507*
E030	5’ GGAATCAATTGGCAGGTCAT 3’	qPCR primer; detection of *bd3507*
E031	5’ GGTACGTTCGACGTTTCCAT 3’	qPCR primer; detection of *dnaK*
E032	5’ TGAGCGGAAGAAAGTTCGAT 3’	qPCR primer; detection of *dnaK*
E042	5’ ACAAAGCAGCCAGAGTGTTC 3’	qPCR primer; detection of *bd1244*
E043	5’ AACGTTGATCACGGTGTTGT 3’	qPCR primer; detection of *bd1244*
E044	5’ AGAACGTCGAACTGCACAAT 3’	qPCR primer; detection of *bd1431*
E045	5’ ATAGGCATAGGCCAGGTTGT 3’	qPCR primer; detection of *bd1431*
E046	5’ AAGCTTACGACAACCGTCTG 3’	qPCR primer; detection of *bd1934*
E047	5’ ACTGGATTTCTGCCCACTTG 3’	qPCR primer; detection of *bd1934*
E053	5’ CAACGCCCTTCACCAGTACT 3’	qPCR primer; detection of *dnaA*
E054	5’ ATTGCGCTTGGAGGATCTCAG 3’	qPCR primer; detection of *dnaA*

Table S2. List of genes encoding predicted non-cytoplasmic DNases in *B. bacteriovorus* HD100.

**Gene**	**Predicted signal peptide (yes/no)**[a]	**Type of signal peptide**[a]	**Predicted localization of encoded protein** [b]
bd0934	Yes	Sec/SPI	extracellular
bd3507	Yes	Sec/SPI	extracellular
bd1244	Yes	Sec/SPI	periplasmic
bd1934	Yes	Sec/SPI	unknown
bd1431	Yes	Sec/SPII (Lipoprotein)	unknown
bd1501	Yes	Sec/SPII (Lipoprotein)	unknown

[a] Presence/type of signal peptide predicted by the SignalP v.5.0 program (3). Sec/SPI - secretory signal peptides transported by the Sec translocon and cleaved by Signal Peptidase I; Sec/SPII – lipoprotein signal peptides transported by the Sec translocon and cleaved by Signal Peptidase II.

[b] Protein localization predicted by the pSORTb program (4)

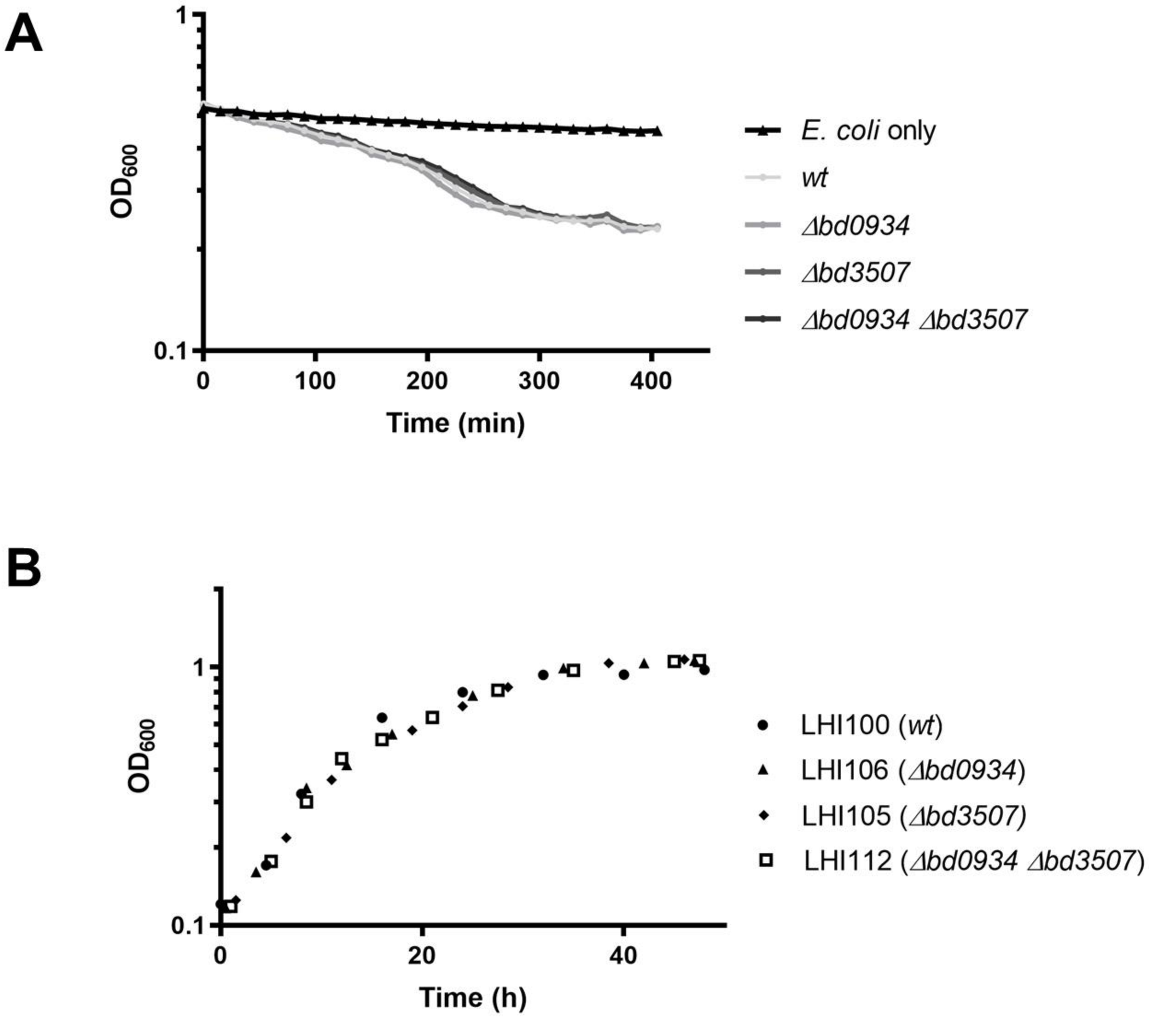

Figure S1. A) Predatory kill curves of *B. bacteriovorus* HD100 (*wt*), LHD104 (*Δbd0934*), LHD103 (*Δbd3507*) and LHD110 (*Δbd0934 Δbd3507). E. coli* DH5α prey was mixed with *B. bacteriovorus* cells at m.o.i. of 3 and incubated at 29°C with aeration. CaHEPES buffer was used in a control culture instead of predator (*E. coli* only). Optical density was measured every 15 min. **B)** Growth curves of host-independent variants of the respective *B. bacteriovorus* strains. Cells were grown in PY broth amended with 3 mM $MgCl_2$ and 2 mM $CaCl_2$ at 29°C with aeration. Optical density was measured every 30 min. For clarity of the graph only selected data points are presented.

Predatory kill curves and growth curves experiments were performed in 3 biological repeats. Data from one representative experiment are shown. Error bars representing standard deviations of a triplicate sample (for predatory kill curves) or pentaplicate sample (for growth

curves) are hidden by the symbols. In growth curve experiments, two individual isolates of each host-independent strain were tested and showed similar growth rates. Thus, for clarity of the graph, data for only one isolate is shown.

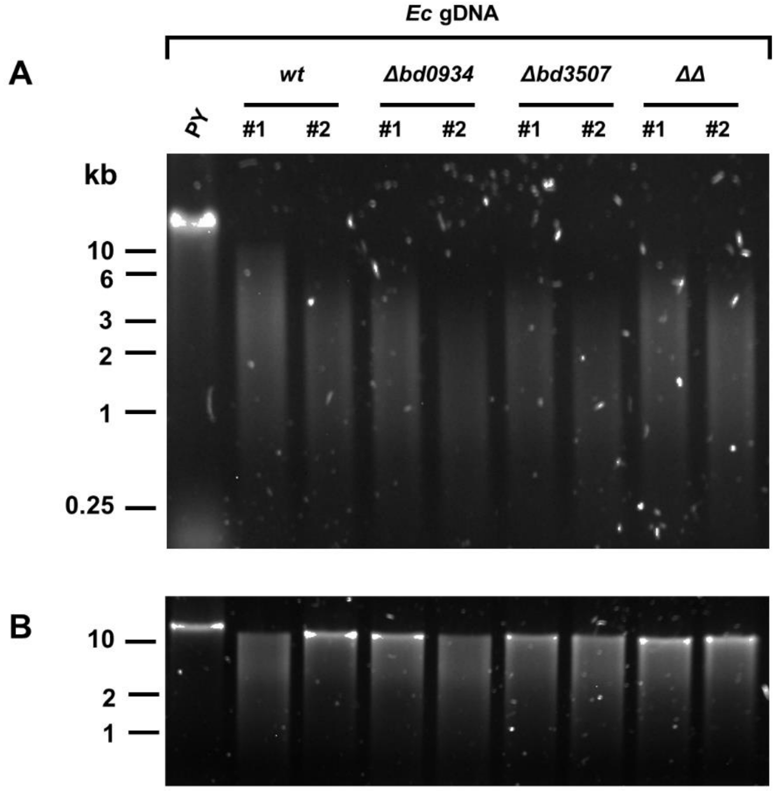

Figure S2. Extracellular DNase activity of host-independent strains. **A)** *E. coli* genomic DNA (40 ng/µl) was mixed with an equal volume of crude supernatant collected from host-independent cultures of the indicated *B. bacteriovorus* strains (*wt* – strain LHI100, *Δbd0934* – strain LHI106; *Δbd3507* – strain LHI105; *ΔΔ* [*i.e Δbd0934 Δbd3507*] – strain LHI112). Two independent isolates of each strain were tested (labelled #1 and #2, respectively). *E. coli* gDNA incubated with PY medium served as a negative control. After 1h incubation at 37°C, samples were analysed by 1 % agarose gel electrophoresis. **B)** Results of the analogous experiment as shown in the panel A, with the following modifications: supernatant was diluted 10 ×, gDNA concentration was 65 ng/µl and the incubation time was reduced to 15 minutes.

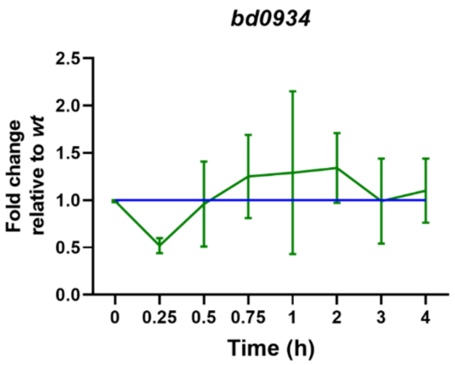

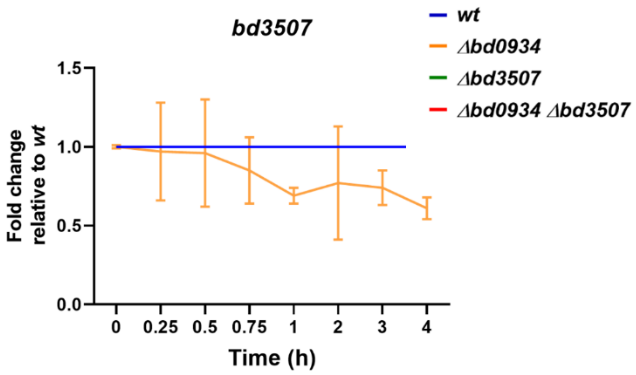

	0m	15m	30m	45m	1h	2h	3h	4h
Δbd3507	-	**** ↓	ns	ns	ns	ns	ns	ns

	0m	15m	30m	45m	1h	2h	3h	4h
Δbd0934	-	ns	ns	ns	**** ↓	ns	*** ↓	**** ↓

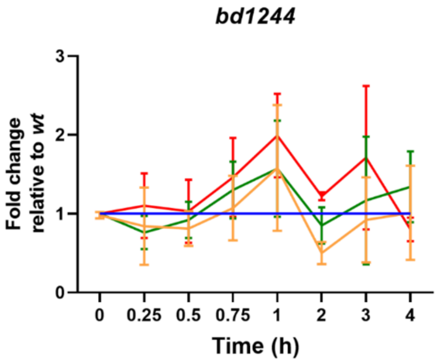

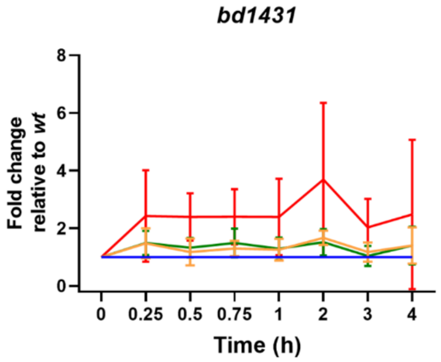

	0m	15m	30m	45m	1h	2h	3h	4h
Δbd0934	-	ns	ns	ns	ns	* ↓	ns	ns
Δbd3507	-	ns	ns	ns	ns	ns	ns	ns
ΔΔ	-	ns	ns	ns	* ↑	* ↑	ns	* ↓

	0m	15m	30m	45m	1h	2h	3h	4h
Δbd0934	-	ns	ns	ns	ns	* ↑	ns	ns
Δbd3507	-	ns	ns	ns	ns	ns	ns	ns
ΔΔ	-	ns	* ↑	* ↑	ns	ns	ns	ns

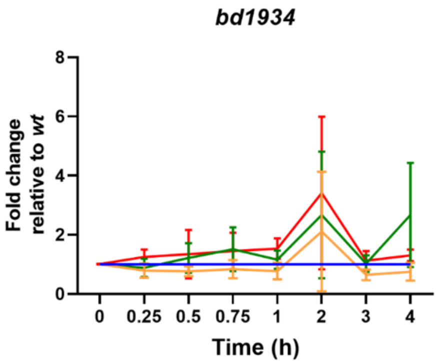

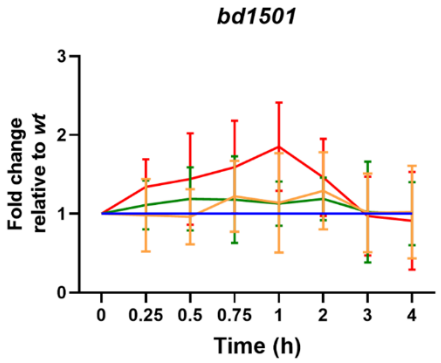

	0m	15m	30m	45m	1h	2h	3h	4h
Δbd0934	-	ns	* ↓	ns	ns	ns	** ↓	ns
Δbd3507	-	ns	ns	ns	ns	ns	ns	ns
ΔΔ	-	ns	ns	ns	* ↑	ns	ns	* ↑

	0m	15m	30m	45m	1h	2h	3h	4h
Δbd0934	-	ns	ns	ns	ns	ns	ns	ns
Δbd3507	-	ns	ns	ns	ns	ns	ns	ns
ΔΔ	-	ns	ns	ns	* ↑	ns	ns	ns

Figure S3. Normalization and statistical analysis of the data presented in Figure 5. For each time point the transcript levels were normalized relative to the wild type strain. Results of both

experiments are combined and are presented as a mean ± SD. The table below each graph shows the results of Student's *t*-test which determined statistical significance with respect to wild type strain at the indicated time point (ns – not significant; * - $p \leq 0.05$; ** - $p \leq 0.01$; *** - $p \leq 0.001$; **** - $p \leq 0.0001$). ↑ - increased transcript level; ↓ - decreased transcript level.

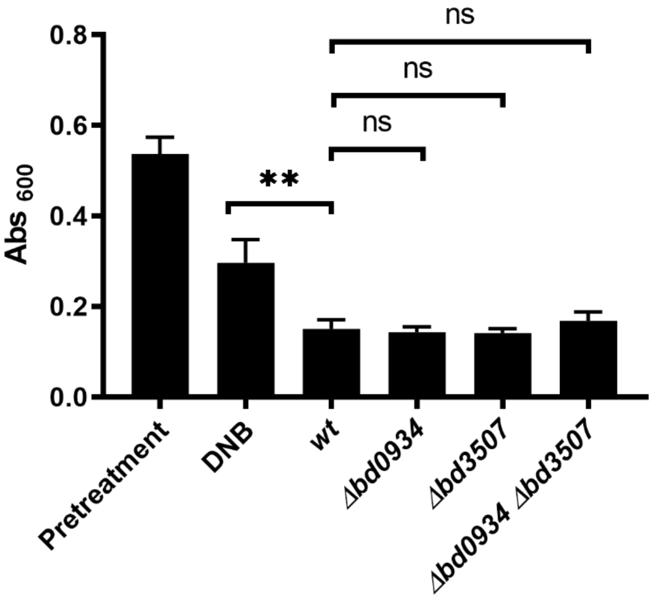

Figure S4. Prey biofilm elimination assay. Indicated *B. bacteriovorus* strains were added to a pre-formed *E. coli* S17-1 biofilm. The amount of biofilm remaining after 24h of incubation was quantified by crystal violet staining and measuring the absorbance at 600 nm. The assay was repeated 3 times. Results of one representative experiment are shown. Error bars represent standard deviations of technical quadruplicates. Student's *t*-test was used to determine statistical significance between the two indicated samples (** - $p \leq 0.01$; ns – not significant). *wt* – strain HD100; *Δbd0934* – strain LHD104; *Δbd3507* – strain LHD103; *Δbd0934 Δbd3507* – strain LHD110; DNB medium was used as negative control.

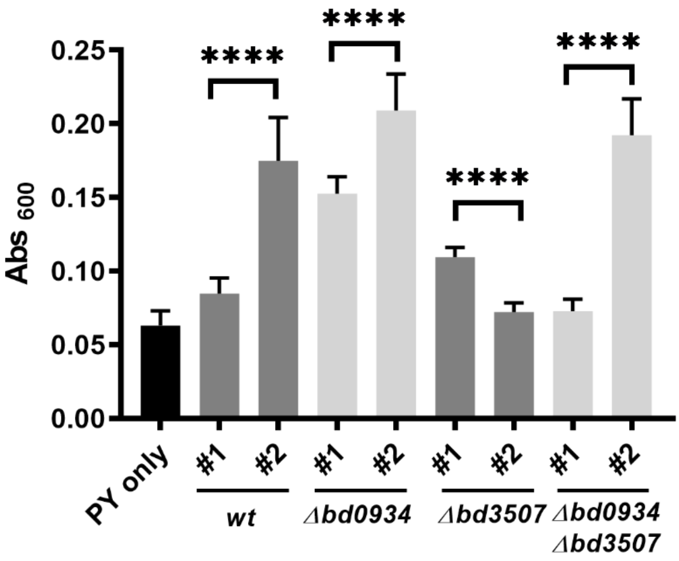

Figure S5. Self-biofilm formation assay by host-independent *B. bacteriovorus* strains. Biofilm was developed in PVC microtiter plates during a 48h period and quantified by crystal violet staining followed by absorbance measurement at 600 nm. Two independent isolates of each strain were tested (labelled #1 and #2, respectively). The assay was repeated 3 times. Results of one representative experiment are shown. Error bars represent standard deviations of 10 technical replicates. Student's *t*-test was used to determine statistical significance between the two indicated samples (**** - $p \leq 0.0001$). *wt* – strain LHI100; *Δbd0934* – strain LHI106; *Δbd3507* – strain LHI105; *Δbd0934 Δbd3507* – strain LHI112; PY medium was used as a negative control.

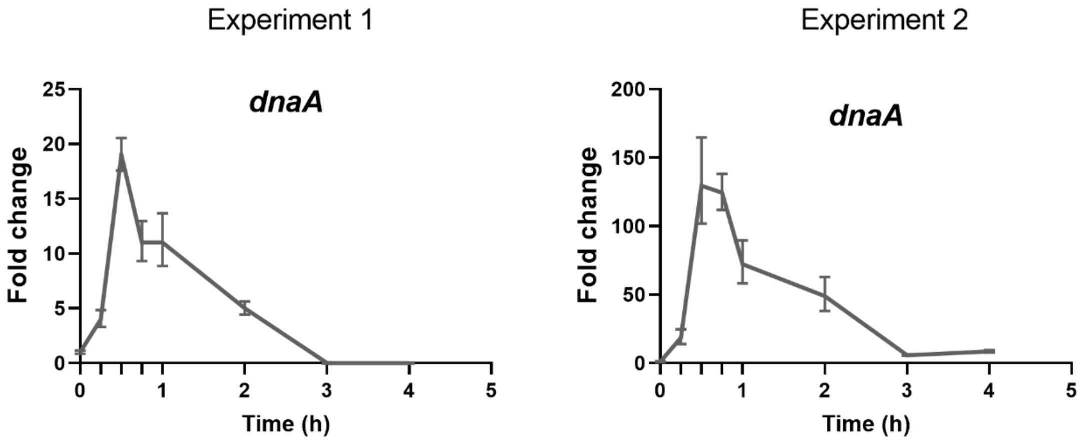

Figure S6. Temporal expression of *dnaA* (encoding chromosomal replication initiator protein DnaA) in *B. bacteriovorus* HD100 strain. RNA was isolated from samples collected at the indicated time points of the predatory cycle. Data obtained from RT-qPCR were analysed using the $2^{-\Delta\Delta CT}$ method. The experiment was repeated 2 times and the results of each experiment are shown in separate panels (Experiment 1 and Experiment 2, respectively). The m.o.i. determined retrospectively for Experiment 1 was 2, and for Experiment 2 was 1. Error bars represent standard deviations of a duplicate sample.

Supplementary References

1. Lambert C, Sockett RE. 2008. Laboratory maintenance of *Bdellovibrio*. *Curr Protoc Microbiol* Chapter 7:Unit 7B.2.

2. Lambert C, Sockett RE. 2013. Nucleases in *Bdellovibrio bacteriovorus* contribute towards efficient self-biofilm formation and eradication of preformed prey biofilms. *FEMS Microbiol Lett* 340:109-116.

3. Nielsen H, Engelbrecht J, Brunak S, von Heijne G. Identification of prokaryotic and eukaryotic signal peptides and prediction of their cleavage sites. *Protein Eng*10:1-6 (1997).

4. Yu NY, Wagner JR, Laird MR, Melli G, Rey S, Lo R, Dao P, Sahinalp SC, Ester M, Foster LJ, Brinkman FSL. 2010. PSORTb 3.0: Improved protein subcellular localization prediction with refined localization subcategories and predictive capabilities for all prokaryotes. *Bioinformatics* 26(13):1608-1615.
